# Prediction of tissue rupture from percolation of local strain heterogeneities for diagnostics

**DOI:** 10.1038/s43856-025-00897-5

**Published:** 2025-05-24

**Authors:** Friedrich Schütte, Sabrina Friebe, Denny Böttcher, Michael Andrew Borger, Madlen Uhlemann, Stefan G. Mayr

**Affiliations:** 1 Leibniz Institute of Surface Engineering (IOM), Leipzig, Germany; 2 Division of Surface Physics, Department of Physics and Earth System Sciences, University of Leipzig, Leipzig, Germany; 3 Faculty of Veterinary Medicine, University of Leipzig, Leipzig, Germany; 4 Department of Cardiac Surgery, Leipzig Heart Center, Leipzig, Germany

**Keywords:** Aneurysm, Biophysical methods, Biophysics

## Abstract

**Background:**

A plethora of medical conditions, ranging from torn ligaments to aneurysmic blood vessels, are caused by failure of mechanically stressed biological tissues until rupture. Clearly prediction of the potential loci of tissue failure prior to rupture is highly desirable for prophylactic measures, preferentially in sufficiently early stages of the disease.

**Methods:**

Mechanical heterogeneities are identified from local mechanical strains obtained from image sequences recorded during uniaxial tensile testing of reconstituted collagen (both, in experiments and finite element model (FEM) calculations) and horse aorta explants, respectively, as well as of the pulsating aorta using magnetic resonance imaging (MRI).

**Results:**

Within this work we present a comprehensive study on the biomechanical concept that percolated local mechanical strain heterogeneities can serve as valid indicators to predict the loci of tissue rupture already from straining behavior within the elastic regime. While we first experimentally validate the predictive capabilities of our strain percolation analysis for reconstituted rat tail collagen fibers and horse aorta explants, we unveil the structural origins of mechanical heterogeneities on the network level using FEM calculations based on digitized confocal laser scanning microscopy (CLSM) measurements. To demonstrate the diagnostic capabilities, we successfully predict potential occurrence and location of an aortic aneurysm in a patient with documented Marfan syndrome from MRI video sequences recorded of the pulsating aorta six years prior to surgery.

**Conclusions:**

Detection of local mechanical heterogeneities and their percolation behavior bears predictive capabilities for tissue failure before it actually has occurred and thus promises large potential for diagnostics and therapy.

## Plain language summary

Numerous medical conditions are related to failure of biological tissues due to mechanical stresses, resulting in tearing and rupture of tissue. We explore whether it is possible to predict tissue rupture before failure occurs. We find that tissue rupture is preceeded by a merging phenomenon of locally mechanically weak regions. Our approach can be applied to data obtained during medical imaging and so could potentially be used clinically in the future.

## Abstract (web summary)

Schutte et al. investigate whether percolated local mechanical strain heterogeneities can predict tissue rupture. They successfully predict an aortic aneurysm in a patient with documented Marfan syndrome from magnetic resonance imaging (MRI) data, showing clinical utility of their method.

## Introduction

Resilience and load-bearing capabilities of soft biological tissues are of life-critical importance in vertebrates^1,2^. Naturally occurring, disease-induced, and iatrogenic defects potentially cause several long-term and even fatal clinical conditions^3,4^. The development of aortic aneurysms is associated with high morbidity and mortality rates, especially in the setting of life-threatening acute aortic dissection or aortic rupture^5,6^. The reported incidence of thoracic aortic aneurysms remains high, with 5.3 per 100,000 individuals per year^6^. Interestingly, aortic dissection occurs predominantly in dilated segments of the aorta^7^. In addition to the highly lethal rupture of aneurysms in the aorta^8–10^, uterine rupture during childbirth^11^, injuries of tendons, ligaments, and skin^12,13^, as well as defects caused by surgeries such as cesarean sections^14,15^ and tissue grafts^16^ are associated with a high risk of subsequent complications. Besides experiments, non-linear continuum constitutive theories have successfully been employed to study deformation and failure of a variety of tissues, including soft tissues^17,18^, meniscal tears^19^, subcutaneous tissues^20^, and very recently cartilage and fibrous tissues^21^. Prediction of the loci, where ruptures are expected to occur (termed “rupture zones", or “RZs" in the following), is still a greatly unresolved challenge even with modern imaging techniques, including sonography as well as MRI and computer tomography (CT)^22–24^. Reliable identification of RZs in an early stage of the disease would certainly prove to be highly beneficial for targeted treatment, e.g., via surgical repair and optimal medical therapy.

Finite deformations in tissues across different time and length scales often show heterogeneous mechanics related to their structural complexity and partially disordered nature (see, e.g., ref. ^25^ and references therein for a recent review). While phenomenological models to account for mechanical response of soft biological tissues mostly on macroscopic scales are certainly successful, a complete scale-bridging understanding is still illusive at this point^26,27^. In disordered non-living matter the concept of shear transformation zones^28–31^, viz. structurally induced spatiotemporal mechanical heterogeneities, has revolutionized understanding in plastic deformation, rheology and fracture mechanics. Inspired by these concepts, we came up with the notion, that tissue “fracture" is a percolation phenomenon of highly yielding mechanical heterogeneities (HYMHs) across the loading direction, constituting the RZ.

Within the present work, we demonstrate that mechanical heterogeneities in reconstituted collagen I networks can reliably be detected and quantified using local strain tensors obtained via digital image correlation (DIC) upon applying a global strain change to a reference sample. HYMHs can then dynamically be defined as mechanical heterogeneities that yield more than a threshold strain. If this threshold strain is chosen as the maximum strain that results exactly in one percolation cluster of HYMHs across the straining direction, we term it “percolation strain", *s*. As we will demonstrate in this manuscript, the corresponding cluster of percolated HYMHs constitutes a suitable prediction for the prospective RZ, viz., the location where tissue rupture is expected to occur at high strains. Employing this approach thus allows for the prediction of the location of collagenous tissue rupture way before it actually occurs.

With the help of finite element calculations on a digitized collagen network, we then track down the physical origin of this heterogeneous behavior, which is found in the density of the network structure. Finally, we transfer and validate our findings first to a more complex tissue, viz., horse aorta explants, and finally give a diagnostic perspective by predicting the potential occurrence of an aortic aneurysm from an MRI video sequence recorded of the pulsating aorta of a patient with Marfan syndrome six years prior to surgery.

## Methods

### Extruded collagen fibers

Reconstituted collagen I fibers were prepared by extruding rat tail collagen type I, as described by us previously^32^. Most importantly, 10 mg/ml solution (Cat.No. 50201, ibidi GmbH, Germany) was first loaded into a syringe, centrifuged at 1000 rpm to extract air bubbles and extruded through PTFE-microtubes with an infusion rate of 0.2 ml/min (controlled by a syringe pump) into a 37 ^∘^C bath of fiber formation buffer (400 ml dH_2_O, 1.7 g phosphate buffer Na_2_HPO_4_, Cat. No. S3264, Sigma-Aldrich, Germany; 2.75 g TES C_6_H_15_NO_6_S, Cat. No. T1375, Sigma-Aldrich, Germany; 3.16 g sodium chloride NaCl, adjusted drop-wise by 5.0 N NaOH to a pH of 7.5). After polymerization within the buffer for 1 h and subsequent stepwise dehydration (by immersion into 100%, 70%, and 30% isopropyl alcohol, respectively, for 1 h each), the fibers were rinsed in destilled water (for 1 h) and air-dried for 20 h. Superglue (medium-viscosity, Cat. No. Z105899, Sigma-Aldrich, Germany) was used to mount the samples onto sample holders for mechanical testing.

After rehydration in distilled water, the wet collagen fibers with a diameter of ~200 μm were crosslinked by applying 10 MeV electrons at a dose of 10 kGy in a bath of distilled water in inert plastic sample holders. In doing so, we follow our protocol for which the detailed mechanistic reaction kinetics have been unveiled and demonstrated to result in the introduction of biomimetic crosslinks into collagen^32,33^.

Tensile testing of the crosslinked fibers was performed using a ZwickRoell testing machine (type Z0.5 TS, ZwickRoell GmbH & Co. KG, Germany). The built-in force sensor (type Xforce HP, ZwickRoell GmbH & Co. KG, Germany) measures up to a nominal force of 5 N and has been calibrated to accuracy class 1 (0.4% of the nominal force, i.e., 0.02 N). The computer software testXpert III sets the parameters and collects the force data for the stress–strain curves. All tensile tests were carried out strain-controlled. The loading rate was uniformly 1.5 mm/min, which corresponds to a strain rate of 0.2 %/s. The measured force acting in the direction of the fiber was converted into stress. The fibers were tested in a custom-designed incubator at a relative humidity of 80 % ± 5% and room temperature.

Shape and surface structure of collagen fibers were recorded with an DinoLite AM7915MZT (AnMo Electronics Corporation, Taipei, Taiwan) light microscope using a frame rate of 10 frames per second (50 frames/% strain). DIC was performed using the software Ncorr^34^. Strain radius and subset spacing were set to 15 and 1, and the norm of the difference vector and maximum number of iterations were set to 10^−6^ and 50, respectively. Local strains were obtained in Green-Lagrangian mode.

### Horse aorta

A specimen of the descending thoracic aorta was taken during autopsy of a 23-year-old German Warmblood stallion. The horse had been euthanized due to suffering from severe colic resulting from rupture of the cecum with subsequent peritonitis; approval by the IRB was thus not required. Equine aorta explants (sample dimensions 7 mm × 3.2 mm × 11.5 mm) were mechanically tested on the same day as autopsy was conducted, i.e., only hours after the horse had been euthanized. Directly after autopsy, the aorta segments were first stored in air-tight plastic bags and then immersed in phosphate-buffered saline to prevent drying during transport and mounting in the tensile tester. Histological examination of the sampled aortic segment as well as histochemical stainings (picrosirius red to visualize collagenoid and elastic fibers, as common in optical pathomorphological routine diagnostics) did not reveal any signs of any appreciable diseases. During tensile testing, the sample was clamped between two jaws, and a double-sided leadscrew drove the sample in the field of view. To increase the contrast for DIC, abrasion from sandpaper was sprinkled on top. The tensile test was carried out strain-controlled using a Microtest (Deben Ltd., Suffolk, U.K.) tensile stage with a 200 N load-cell at a loading rate of 6 mm/min (strain rate 0.78%/s) and control software to capture the force data with a frequency of 10 Hz. Simultaneously to the stress–strain experiment, the equine aorta surface was recorded with an IDS camera system (UI-3240MB-C-HQ, IDS Imaging Development Systems GmbH, Obersulm, Germany) and a FUJINON objective (HF16HA-1B, Fujifilm, Tokyo, Japan) to obtain a set of images for DIC (20 fps, corresponding to 25 frames/% strain). Strain radius and subset spacing during DIC were set to 30 and 3, and norm of the difference vector and maximum number of iterations were set to 10^−4^ and 100, respectively.

### MRI scans of human aorta

MRI scans of the aortic root and ascending aorta of a 43-year-old patient with documented Marfan syndrome were routinely recorded in 2014 and 2020 according to established clinical practice to screen for aortic aneurysms^35^. The data was obtained in an anonymized way and analyzed in the same way as the other samples reported within this manuscript. According to a statement by the local ethics board of the Faculty of Medicine at the University of Leipzig, Germany, our study constitutes a case report, i.e., a retrospective description of medical therapy; in particular, it is not a prospectively planned research project. For that reason, no ethics approval is required. Regarding personal medical data protection, however, all MRI data was completely anonymized, and the patient declared consent to the publication of the data included in the manuscript.

### Percolation analysis

**Fig. 1:**
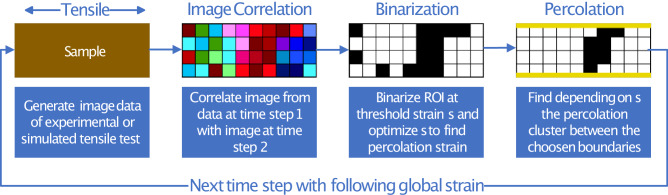
Scheme of the steps performed to determine percolating clusters of local strains upon application of global tensile strain: Geometric constraints of the boundaries can be adjusted depending on the tensile direction and percolation orientation.

An algorithm was developed to study the strain percolation in the presented collagenous systems. For the percolation analysis, the strain matrix resulting from the strain tensor from DIC was considered as a square lattice. Two grid squares are connected if they are nearest neighbors. A percolating cluster is defined as a set of connected and occupied lattice sites that extend from one side to the opposite side of the region under consideration. The system is percolating if there is a percolating cluster in the system. Percolation was studied in 2D using the strain matrix *E*_*xx*_ from applying the software Ncorr^34^, which maps the strains in the tensile direction. The percolation was studied in the region of interest investigated via DIC analysis. For the experimentally obtained data, the region of interest was defined as the entire image area in which the surface of the samples is mapped. In the simulation, the local strains from the modeled cell were considered. The procedure for analyzing a single time step of a tensile test and continuing is shown in Fig. 1.

**Fig. 2:**
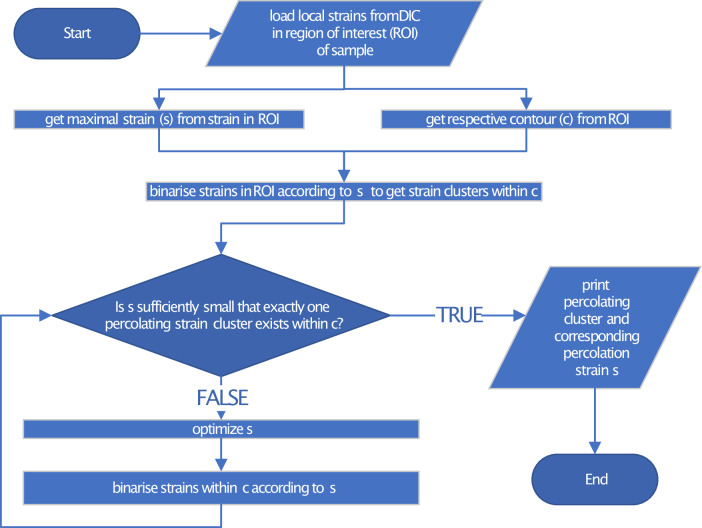
Flow diagram for determining the percolation strain s: It is defined as the smallest local strain where exactly one percolating cluster with values higher than s forms in the ROI of the sample. Local strains were determined by DIC in the ROI. Depending on the boundaries between which site percolation is to be investigated, the respective contours can be considered in the direction of tension. Furthermore, only a single strain matrix from one image is analyzed. For a set of images, such as a video with a time series of a tensile test, it is still necessary to loop over the entire sequence. Note: conceptually, as specified here, the routine can only be performed if a percolating cluster of positive strains exists. Details of single processes were omitted for simplicity.

For clustering, the strain matrix was binarized. Below the initially set threshold strain, which corresponds to the maximum strain in the entire matrix, strains were set 0. At and above the threshold, strains were set 1. This allowed clusters to form in the region of interest. Each cluster and all of its respective grid locations were assigned a number so that the numbers of the clusters and the clusters themselves could be distinguished. The lattice sites of the edges of the region of interest were also successively set with the same numbers as those of the clusters, while it was checked whether an overlap of the edges, which were in the tensile direction, was formed with the resulting clusters and which cluster, if any, had an overlap. If there were fewer or more than one percolating cluster, the threshold was adapted using an optimization routine. The termination criterion of this routine was met if the change was less than 0.1% between the previous and the current threshold strain. If only one percolating cluster was left and the termination criterion was met, this cluster was set as the percolating cluster with the corresponding threshold strain called the percolation strain. The percolation strain is a sufficiently small strain such that at least one strain cluster with local strains at or higher than the percolation strain percolates in the contour regions that are parallel to the tensile direction. The flowchart in Fig. 2 summarizes these operations for a single time step.

The percolation strain could be determined for a series of global strains, i.e., for a sequence of images showing a tensile test of the sample. In addition, the position of the percolating cluster as a function of the applied global strain was investigated, as well as the cluster size. Further geometric properties of the clusters can be read out.

### Finite element modeling of digitized collagen networks

#### Determination of network topology

Confocal Laser Scanning Microscopy (CLSM) measurements of reconstituted collagen I networks, as prepared from a mixture of collagen I monomers from rat tail (Collagen R, 0.4% solution, Cat. No. 47256.01; SERVA Electrophoresis, Germany) and bovine skin (Collagen G, 0.4% solution, Cat. No. L 7213; Biochrom, Germany) in a mass fraction of 1:2, were taken from a previous study performed within our group^36^. The topology of experimental collagen networks, in particular the connectivity that the fibrils establish between the vertices, is analyzed and digitized using the ”FIRE” tool^37,38^ for MATLAB^39^. As central output, these routines yield the connectivity between the vertices, which we evaluate regarding the average coordination number between the vertices. The latter, in fact, constitutes the key quantity regarding network stability within the Maxwell connectivity criterion^40^, which provides a threshold (6 in three dimensions) above which the lattice would be stable considering only central forces (i.e., disregarding bending rigidity). Applying this approach for our collagen network yields an average coordination number of 3.95, which implies that fibril bending will dominate the low-strain regime, while a transition to a fibril-stretching-dominated regime is expected at high strains due to alignment of the fibrils along the strain direction. Furthermore, the obtained “digitized” network topology is used as the basis for extensive finite element modeling using Simo-type largely deforming beam elements^41^, as described in the following.

#### Finite element modeling

Macroscopic deformation and fracture of reconstituted collagen networks upon external application of tensile stresses are governed by the mechanics at the network and fibril levels. To obtain an in-depth understanding across length scales and unravel the physical foundations of mechanical heterogeneities and their relation to fracture behavior, we employ finite element calculations. For the sake of reproducibility, we base our modeling on the open-source code “code aster”^42^ for obtaining a grasp of deformation in “digitized” experimental collagen networks on the fibrils scale. The network topologies, as extracted and digitized from CLSM-Stacks (described above) were appropriately converted using awk skripts and meshed using wire discretization within SMESH^43^. Convergence studies showed that a FEM-meshing with 15 FEM-segments per collagen network segment proved to be dense enough to obtain meaningful results, which was subsequently employed in all studies presented here. We employ non-linear static analysis using elastic beam elements with large displacements and rotations, due to Simo et al.^41,44,45^ to realistically account for collagen network deformation due to an applied tensile load. Assuming that the network is composed of collagen fibrils of spherical cross-section (radius 1 μm) with Young’s modulus and Poisson ratio of 1 GPa and 0.35, respectively, we solve the non-linear static problem upon applying a global spatially fixed load with the MUMPS^46^ solver. Displacements as well as fibril stress levels at the FEM-nodes are determined and recorded as a function of externally applied global stress. While the key quantities—displacements and stresses–are thus available within the loci of FEM-nodes, viz., reflect the mechanics on the level of the collagen fibril network, mapping of these quantities onto fields in 3D space is highly desirable. This particularly holds true for the displacement field,

1 $$\vec{u}(\vec{r})\,\,\,\text{with}\,\quad \vec{r}=\left(\begin{array}{c}{x}\\ {y}\\ {z}\\ \end{array}\right),$$

which is related to the Green-Lagrange strain tensor field via

2 $${E }_{ij}\left(\vec{r}\right)=\frac{1}{2}\,\left(\frac{\partial {u}_{i}}{\partial {x}_{j}}+\frac{\partial {u}_{j}}{\partial {x}_{i}}+\frac{\partial {u}_{k}}{\partial {x}_{i}}\frac{\partial {u}_{k}}{\partial {x}_{j}}\right)$$

where we utilize the convention to sum over indices that appear twice within products. We presently determine $\vec{u}(\vec{r})$ by extrapolating the nodal displacements, ${\vec{u}}_{\alpha }$, where *α* denotes an arbitrary FEM-node, to continuous 3D space by employing Gaussian weights, viz.

3 $${\vec{u}} ({\vec{r}}) = \frac{ {\mathop{\sum}\nolimits_{\alpha}} {\vec{u}}_{\alpha} \cdot {e}^{-\left({\left({\vec{r}}-{\vec{r}}_{\alpha} \right)}^{2} / \left(2 {\sigma}^{2}\right)\right)}} {\underbrace{{{\sum}_{\alpha}} {e}^{-\left({\left({\vec{r}}-{\vec{r}}_{\alpha}\right)}^{2} / \left(2 {\sigma}^{2}\right)\right)}}_{={(2\pi)}^{3/2} {\sigma}^{3} \rho({\vec{r}})}}$$

where ${\vec{r}}_{\alpha }$ and *σ* denote the position vector of node *α* and the smearing width (standard deviation of Gaussian smearing). The latter has to be chosen carefully, i.e., as small as possible, but large enough to yield a well-defined displacement field at arbitrary coordinates $\vec{r}$. Within our current work, we found that *σ* = 4.4 μm proved to be a reasonable choice. It is worth mentioning within this scope, that the denominator of Equ. (3) constitutes a simple measure of the fibril density, $\rho \left(\vec{r}\right)$. We employ the TOMS661 library^47^ to determine the first derivatives of $\vec{u}(\vec{r})$ that are necessary to calculate the Green-Lagrange strain fields in 3D, ${E }_{ij}\left(\vec{r}\right)$ (Equ. (2)); 3D fields are discretized on 512 × 512 × 512 grids throughout.

### Statistics and reproducibility

The code for the percolation analysis, as well as evaluation routines, were developed in Python 3.8. For all samples investigated, the algorithm described above yielded statistically significant predictions. All statements made in this manuscript were checked for statistical significance and are representative of the described set of samples. Nevertheless, application in diagnostics requires a comprehensive clinical study, which is currently in preparation.

### Reporting summary

Further information on research design is available in the Nature Portfolio Reporting Summary linked to this article.

## Results and discussions

### Rupture of reconstituted collagen I fibers

**Fig. 3:**
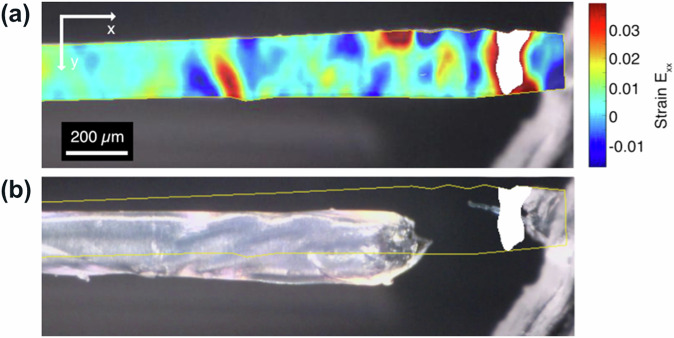
Rupture behavior of energetic-electron-crosslinked reconstituted collagen I fibers: **a** Reference image at a global Green-Lagrange strain of *ϵ* = 40.4% and color-coded Green-Lagrange strain field *E*_*xx*_ for globally straining from *ϵ* = 40.4% to 41.4%. The predicted RZ is drawn in white. **b** Ruptured fiber and RZ, as predicted before rupture. The contour of the fiber at an elongation of 41.4% is sketched in yellow.

We first focus on reconstituted collagen I fibers that are composed of a homogeneous and isotropic fibril network due to gentle extrusion, followed by biomimetic energetic-electron induced crosslinking using a dose of 10 kGy^32,33^. Tensile tests (Supplementary Fig. 1) were carried out in the axial direction (aligned along the *x* axis of our coordinate system) until rupture, while global stresses as well as an optical microscopy image sequence of the deforming sample surface were recorded. Rupture occurred at a critical Green-Lagrange strain and First Piola-Kirchhoff stress of *ϵ*_*c*_ = 45.33% and *σ*_*c*_ = 4.8 MPa, respectively, in a ductile manner (ductility results from energetic-electron assisted crosslinking^32^), as shown in Fig. 3.

After finishing the experiments, local Green-Lagrange strain tensor fields (the component *E*_*xx*_ denotes local “relative length change" along the x direction) were determined from the videos of the experiment. Figure 3 exemplarily shows the results for globally straining from *ϵ* = 40.6% to 41.6%. It is striking to see that long before rupture occurs, higher than average local strains *E*_*xx*_ are detected in those regions of the sample, where later rupture, in fact, occurs. To pin things down even more, rupture seems to proceed within a percolating cluster of material, which mechanically yields above average, i.e., more than a threshold strain *s* (the definition of *s* strictly defines HYMHs), long before rupture actually has occurred. If we choose for *s* the maximum strain level, which just results in one percolating cluster of HYMHs, so that the percolating cluster completely traverses the sample from one side to the other across the straining direction, we term this particular *s* “percolation strain". This cluster then constitutes the predicted RZ.

Figure 3 shows this scenario for a percolation strain of *s* = 4.3%. It has to be noted that the percolation strain is related to the reference configuration, which is somewhat arbitrarily chosen here as *ϵ*  = 40.4%. That is, depending on the selected reference, the particular level of percolation strain *s* varies. The predicted RZ only slightly fluctuates in shape and position with an increase in global strains (mind that due to the application of Green-Lagrange strains, the predicted RZs are mapped into the reference configuration of the sample). As visualized in Fig. 3, the predicted RZ furthermore perfectly coincides with the actual position of collagen network rupture.

### Structural origins of heterogeneous deformation and rupture in collagen I

**Fig. 4:**
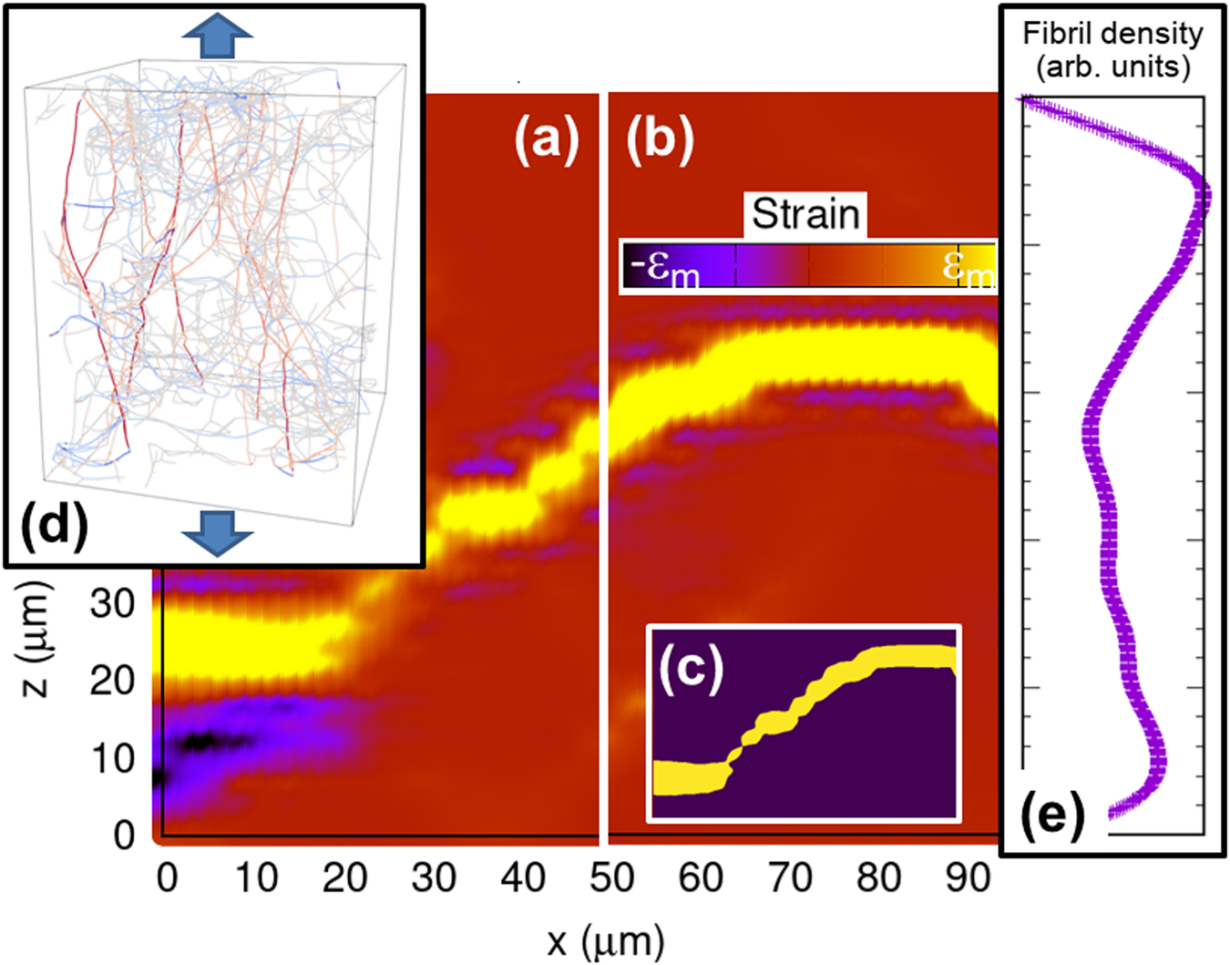
Analysis of percolating local strain heterogeneities, as predicted by a FEM fiber model based on digitized CLSM measurements of collagen I. Spatially resolved Green-Lagange strain, as predicted by FEM calculations for global strains of 0.5% (**a**) and 17% (**b**) and exemplarily shown on the x-z sample surface (*ϵ*_*m*_ = 5% and 150%, respectively). From the beginning of deformation, high strains are concentrated in regions that do not significantly shift within the sample during global straining, as identified by a percolation analysis (**c**). **d** Network topology and fibril stress levels (color-coding using the temperature scheme) exemplarily shown for global strains of 17%. A low fibril area density through (generally curvy) cross-sections across the straining direction dictates both increased local strains and rupture tendency (**e**).

To unravel the structural origins behind heterogeneous mechanics and rupture, we performed FEM calculations on the non-linear deformation mechanics of a digitized reconstituted collagen I network, measured with CLSM previously^48^. Clearly, global deformation mechanics (stress-strain curve–Supplementary Fig. 4) reveals key signatures also observed experimentally, particularly exponential dependence of stress on strain^49^. This global deformation response is mediated by local strains on the network level, which prove to be highly non-affine by revealing mechanical heterogeneities (Fig. 4a, b). HYMHs identified in the very early linear elastic regime keep their character as “being prone to rupture" throughout the deformation process until rupture (which occurs at 17% strain here).

Regarding the physics behind this observation, we first note that HYMHs are characterized by a lower network density or—to be more accurate—a lower than average area density of fibrils passing through the (generally curvy) crossections across the straining direction (Fig. 4e). The fibrils passing through these low density cross sections not only bear the complete mechanical load, but are part of “continuous fibril paths" that branch into the regions of higher network density and range from one end of the sample to the other along the loading direction. When global strains are applied, local stresses are concentrated along these “continuous fibril paths" (visible in red due to color-coding in Fig. 4d), while maximum stress concentration and yield clearly occur within the lowest density cross sections until rupture. That is, higher local strain and rupture are both manifestations of an increased stress concentration due to a lower network density across the straining directions, viz., have the same structural origin. This allows, vice versa, to predict rupture based on local strains long before it actually occurs.

While we have developed this picture for a random collagen network, we expect the concept to be transferable in its basic framework also to more complex networks and tissues. That means, rupture is triggered by percolation of topological weak links of the biopolymer/extracellular matrix (ECM)/tissue network, which reveal their fingerprint already at strains as low as 0.5% in the linear elastic regime. This constitutes the hypothesis for the following.

### Rupture of equine aorta explants

As demonstrated above for reconstituted collagen (which can be regarded as a model system for ECM and tissue), the potential loci of rupture can reliably be predicted by a percolation analysis of HYMHs across the straining direction upon applying strains as tiny as 0.5%, i.e., far within the linear elastic regime. These findings raise hopes that these concepts could potentially be useful in medical diagnostics. As a highly relevant example to validate this hypothesis, we chose aortic aneurysms, which are largely asymptomatic extensions of the aortic diameter and intrinsically bear the risk of spontaneous fatal rupture. According to current clinical practice guidelines, surgeries are performed based on an aortic diameter criterion, which is certainly highly successful, but potentially could be supplemented by a prediction approach, such as ours, regarding the potential rupture site.

**Fig. 5:**
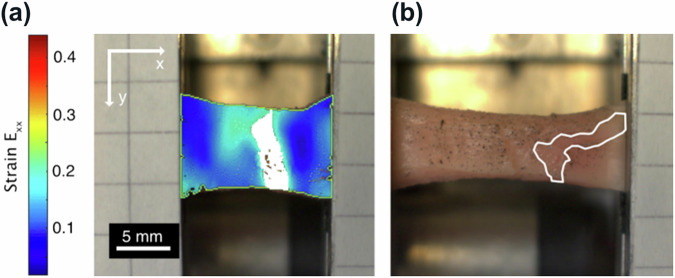
Rupture behavior of equine aorta explants: **a** Equine aorta sample mounted in the tensile stage at 10.45% global strain with predicted RZ in white and contour of region of interest from DIC (light green line). **b** Stretched equine aorta after rupture of the intima (white contour, corresponds to actual RZ) at 63.805% global strain.

As a first step towards this direction, we focused on equine aorta explants, which were mounted with their circumferential direction along the x-axis of the coordinate system in the tensile tester. They were then macroscopically strained, while the First Piola–Kirchhoff stress and an image sequence of the deforming intima were recorded until rupture occurred—for the presented explant at *ϵ*_*c*_ = 109%. The obtained stress–strain curves shown in Supplementary Fig. 2 are consistent with two previous reports^50,51^. Applying the same algorithm as for the reconstituted collagen fibers, predictions for the RZs were obtained. As an example, the predicted RZ obtained for a global and percolation strain of *ϵ* = 10.45% and *s* = 19.85%, respectively, is shown in Fig. 5, taking the unstrained sample as reference. While the predicted RZ fluctuates slightly in shape and position upon further straining, the prediction of the actual RZ of the aortic intima becomes more accurate, the closer the global strain reaches the rupture strain. Nevertheless, when transformed back into the reference configuration, the actual RZ overlaps reasonably well with the predicted RZ (see Fig. 5). Deviations are assumed to originate from i) the low global strain level at which the prediction for the RZ was performed in Fig. 5, ii) the more complex, layered structure (intima, media and adventitia) of aortic tissue, of which only the intima was imaged.

### Mechanical instabilities and aneurysm in human aortas

The Marfan syndrome^35^ constitutes a genetic autosomal-dominant connective tissue disorder which is accompanied by an increased risk for developing an aortic aneurysm and aortic dissection due to a disturbance of the ECM design by fibrillin-1 gene mutations, resulting in mechanical instability^52^. For that reason, it is established clinical practice to screen patients with Marfan syndrome for aortic aneurysm on a regular basis ^35^. For the present study, an anonymized 43-year-old patient with documented Marfan syndrome was screened for an aortic aneurysm in 2014 using an MRI scan—with normal diameters of the aortic root und ascending aorta. As recommended in current guidelines^53^, aortic imaging was performed at regular intervals at the Leipzig Heart Center. In contrast, a second screening in 2020 using MRI again resulted in the detection of an aneurysm of the ascending thoracic aorta. This medical history gives us the unique opportunity to check in retrospect, if our approach to identify potential RZs from percolation of HYMHs is capable of predicting the development of an aortic aneurysm already from vascular imaging data in 2014, where traditional screening was unable to do so. Within this context, it is noteworthy that blood vessels are a highly suitable field for applying our analysis approach, as the fluctuating pressure due to the pulsating heart intrinsically ”performs in-vivo mechanical tests” along the circumferential direction, just as our tensile stage does with aortic explants.

**Fig. 6:**
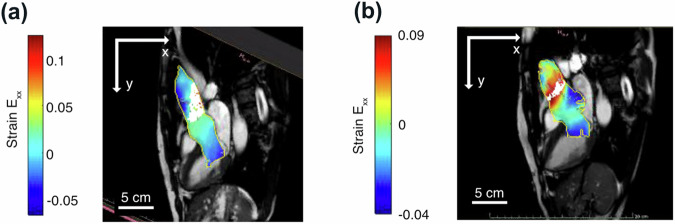
Pulsating ascending thoracic human aorta with color-coded Green-Lagrange strains *E*_*xx*_ (which are largely directed along the circumferential direction) and predicted RZ in white. The contour of the region of interest from DIC is colored in yellow—**a** 2014 vs. **b** 2020.

In Fig. 6 we apply our presented analysis algorithm to an MR image sequence of a pulsating ascending thoracic aorta (20 frames per heart cycle)—recorded in a) 2014 and b) 2020, respectively. Evaluation of the local straining behavior during a pulse cycle results in predictions for the RZ, as drawn in white in Fig. 6a, b, respectively. Strikingly, based on the MRI data of 2014, we predict an RZ in the location where a significant increase in the aortic diameter was seen in the MRI scan in 2020. Thus, our HYMH percolation model is able to identify a potential location of the aortic aneurysm six years prior to its occurrence. In 2020, on the other hand, the predicted RZ has relocated towards the sinotubular junction/aortic root, presumably due to changes in the consistency of the aortic tissue. This prediction is in line with the medical diagnosis obtained in 2020 based on an aortic diameter criterion, which resulted in the decision to perform surgical replacement of the aortic root and ascending aorta.

## Conclusions and outlook

To wrap things up, we have demonstrated using i) reconstituted collagen as an ECM model, ii) FEM-based computer modeling, and iii) equine aorta explants strained ex vivo, that HYMHs play a key role in rupture behavior in biological tissues. Most importantly, using these model systems, i)–iii), tissue rupture was demonstrated to be preceded by a percolation of HYMHs across the straining direction; the location of rupture was found to coincide with the position of the percolation phenomenon on the sample. Strikingly, the percolation phenomenon occurred in all samples investigated already within the elastic regime, which implies that the location of the fracture can be predicted based on reversible straining. As a first test for the potential application of this concept in diagnostics, this approach was successfully applied to a 2D MRI image sequence of the pulsating ascending thoracic aorta of a patient with Marfan syndrome to successfully predict the location of a future aneurysm, which had been examined six years prior to surgery. These initial findings are very promising and might lead to important clinical benefits, because earlier detection of aortic aneurysm with optimized medical care could improve prognosis and long-term survival of this patient cohort at risk.

While our 2D strain percolation analysis in the global straining direction was already highly successful for suitable geometries, we plan, as an outlook, to perform a fully three-dimensional percolation analysis on the local principal strains/invariants of the local strain tensors, which surely will increase applicability and reliability. In this regard, we are currently in the process of preparing a comprehensive clinical study to quantify predictive capabilities by employing a larger-scale statistical approach. In addition, generalization to even other “actively stretched” tissues within living organisms, such as tendons and ligaments, will be highly interesting in the future.

## Supplementary information

Supplementary Information

Description of Additional Supplementary Files

Supplementary Video 1

Supplementary Video 2

Supplementary Video 3

Reporting Summary

## Data Availability

All experimental and modeling data are available from the authors at reasonable request.

## Code availability

The codes employed within the manuscript will be made available upon request to the corresponding author. They were provided to the referees during the peer review process. The code is not being made publicly available at this time because it is currently under active development, and we, as code authors, are employed by the Leibniz Institute of Surface Engineering (IOM) and the University of Leipzig. Thus, the code is the property of these institutions.
