# Supplementary material for: Prediction of tissue rupture from percolation of local strain heterogeneities for diagnostics
Source: Commun Med (Lond). 2025 May 24;5:197. doi: 10.1038/s43856-025-00897-5 (PMC12103514; doi:10.1038/s43856-025-00897-5)
Supplement: Supplementary file 1 — Supplementary Information [file 43856_2025_897_MOESM1_ESM.pdf]

Prediction of tissue rupture from percolation of local strain heterogeneities for diagnostics

Friedrich Schütte[1,2], Sabrina Friebe[1,2], Denny Böttcher[3], Michael Andrew Borger[4], Madlen Uhlemann[4†] and Stefan G. Mayr[1,2*†]

[1]Leibniz Institute of Surface Engineering (IOM), Permoserstr. 15, 04318 Leipzig, Germany.
[2]Division of Surface Physics, Department of Physics and Earth System Sciences, University of Leipzig, Linnéstr. 5, 04103 Leipzig, Germany.
[3]Faculty of Veterinary Medicine, University of Leipzig, An den Tierkliniken 33, 04103 Leipzig, Germany.
[4]Department of Cardiac Surgery, Leipzig Heart Center, Struempellstraße 39, 04289 Leipzig, Germany.

*Corresponding author(s). E-mail(s): stefan.mayr@iom-leipzig.de;
†Shared last authorship. These authors contributed equally to this work.

Supplementary Note

Extruded collagen fibers

A MP4-movie of the tensile test is included as supplementary file.

Linear fitting of Figure 1 resulted in a Young's modulus of 8.19 ± 0.37 MPa ($R^2 > 0.41$) in low strain region up to 10% strain. Due to the sinusoidal increase of the mechanical deflection by means of a fine screw on the ZwickRoell testing machine, the behaviour of the original strains is not strictly linear, which is reflected in the DIC. A strain of 1% corresponds to a deflection of 20 µm.

Horse aorta

A MP4-movie of the tensile test is included as supplementary file.

The stress-strain curve resulting from a tensile test shows a largely linear behaviour up to the rupture point (see Figure 2) with a Young's modulus of 0.291 ± 0.1 MPa ($R^2 > 0.99$). The imaging of the horse aorta surface in parallel with the stress-strain experiment provided a set of images for DIC. The percolating cluster size shows a minimum at $\approx 12.1\%$, which indicates a concentration of local strain is at a maximum here (see Figure 3). Plastic behaviour may occur from this point onwards; the stretching process is supposed to be irreversible.

Fibril network model

A MP4-movie of the stress-coded fibril model is included as supplementary file.

Supplementary Figures

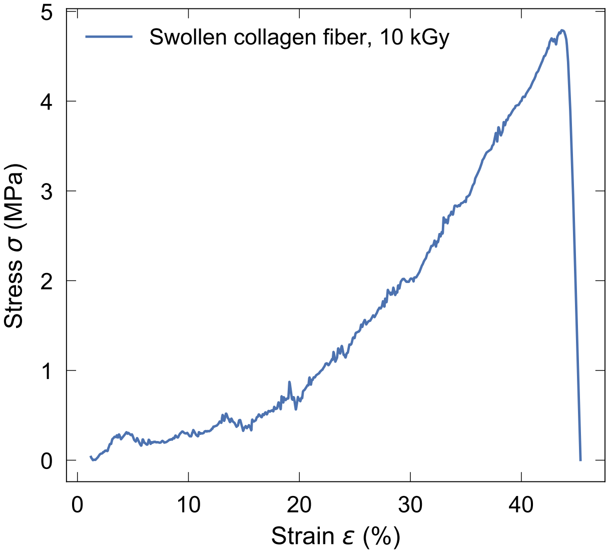

Fig. 1 Stress-strain curve from tensile testing of a energetic electron crosslinked hydrated (swollen) collagen fiber (electron dose: 10 kGy, diameter 200 μm). The force offset due to the experimental setup was removed manually.

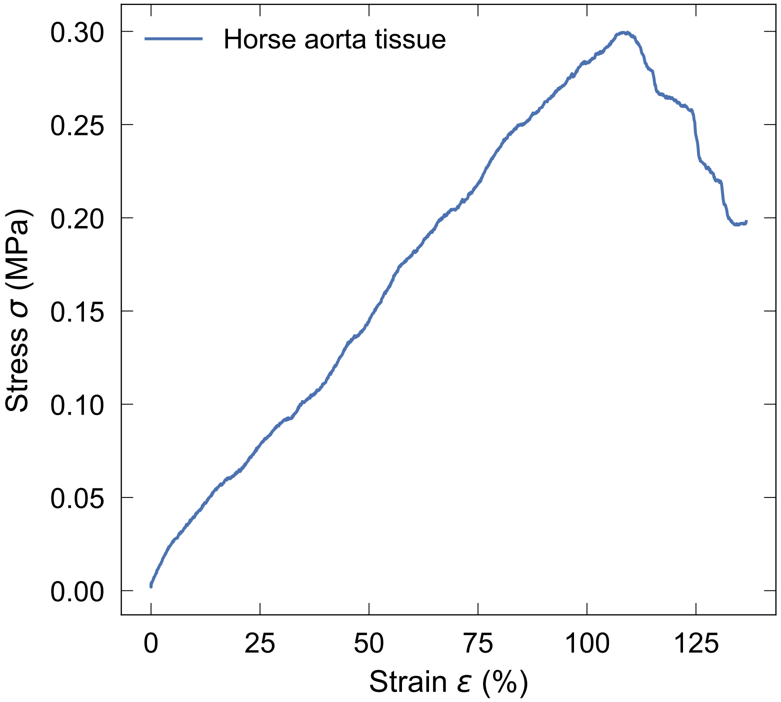

Fig. 2 Stress-strain curve of a horse aorta explant until rupture.

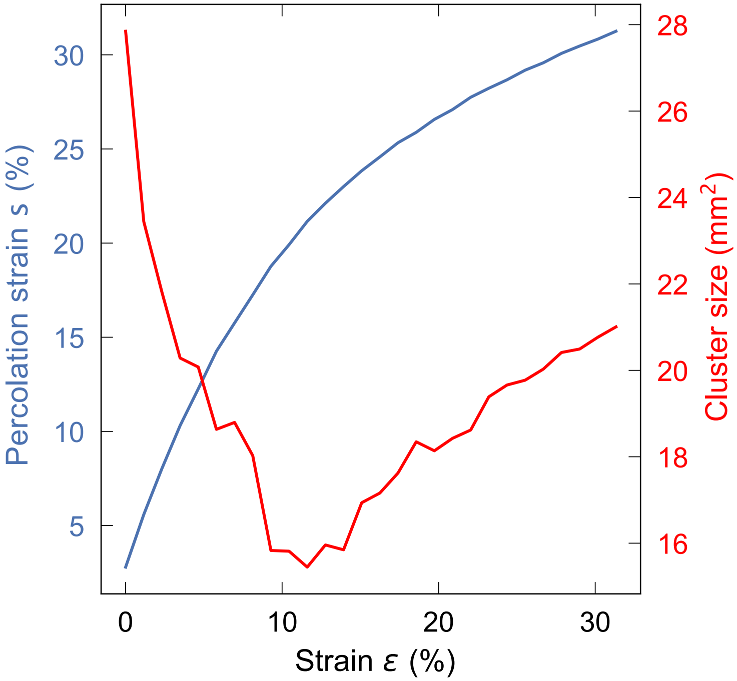

Fig. 3 Percolation strain, s, and size of the percolation cluster for increasing global strains, ϵ, as determined upon evaluation of tensile stretching behavior of a horse aorta explant.

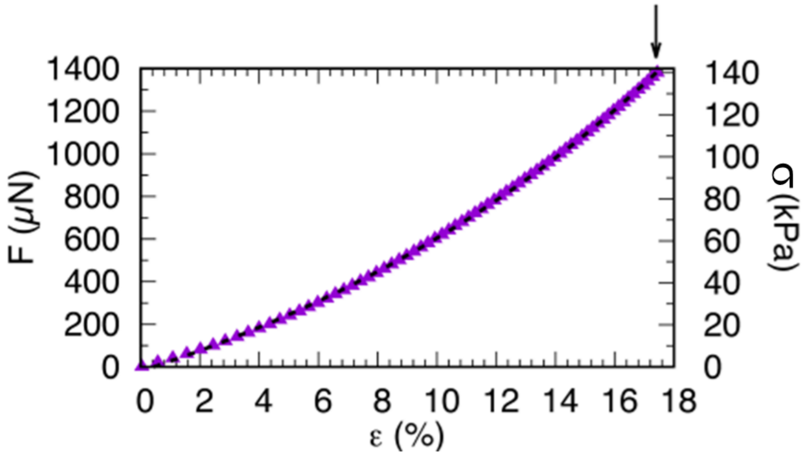

Fig. 4 Green-Lagrange strain vs. force / First Piola Kirchhoff stress σ curve, as predicted by FEM calculations - together with an exponential fit to the data (dashed line). The arrow indicates the strain level that is exemplary discussed in more detail.

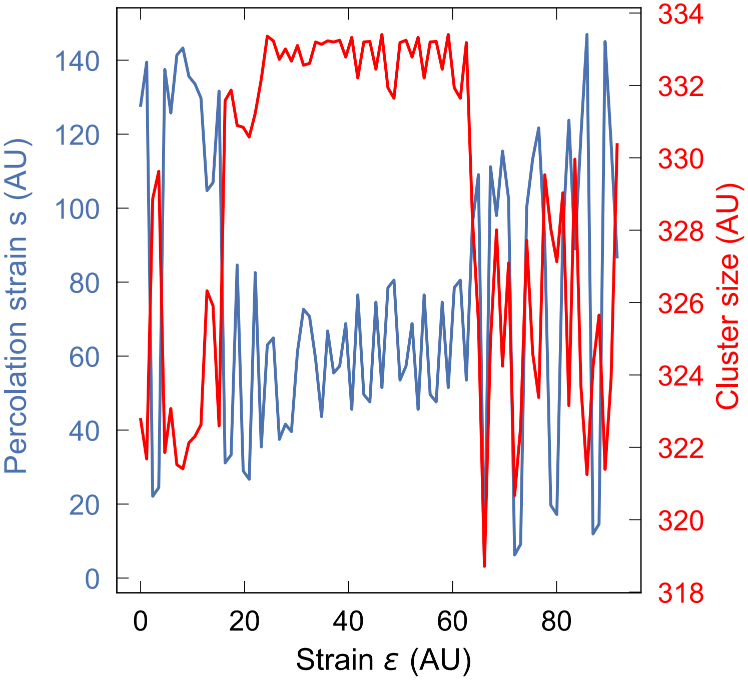

Fig. 5 Percolation strain, s, and size of percolating cluster (measured in arbitrary units, AU), as function of global strain ϵ, for the fibril network model.
