# Supplementary material for: Prediction of tissue rupture from percolation of local strain heterogeneities for diagnostics
Source: Commun Med (Lond). 2025 May 24;5:197. doi: 10.1038/s43856-025-00897-5 (PMC12103514; doi:10.1038/s43856-025-00897-5)
Supplement: Supplementary file 2 — Description of Additional Supplementary Files [file 43856_2025_897_MOESM2_ESM.docx]

Supplementary Video 1 contains Deformation of a digitized collagen network, as predicted by finite element modeling. Fibril stresses are color coded.

Supplementary Video 2 contains Tensile test of an equine aorta explant (example)

Supplementary Video 3 contains Tensile test of an extruded energetic electron crosslinked collagen fiber (example)
